# Evaluation of sites of velopharyngeal structure augmentation in dogs for improvement of velopharyngeal insufficiency

**DOI:** 10.1371/journal.pone.0212752

**Published:** 2019-02-25

**Authors:** Emiko Tanaka Isomura, Kiyoko Nakagawa, Makoto Matsukawa, Ryou Mitsui, Mikihiko Kogo

**Affiliations:** 1 First Department of Oral and Maxillofacial Surgery, Osaka University, Graduate School of Dentistry, Suita City, Osaka, Japan; 2 Unit of Dentistry, Osaka University Hospital, Suita City, Osaka, Japan; Navodaya Dental College and Hospital, INDIA

## Abstract

**Background:**

Velopharyngeal structure augmentation methods are used as alternatives to velopharyngeal plasty. Anatomic sites of implantation/injection vary widely due to a lack of standardized criteria. Here, we experimentally investigated optimal sites of velopharyngeal structure augmentation via saline injection in dogs as they naturally exhibit velopharyngeal insufficiency (VPI).

**Methods:**

Velopharyngeal structure augmentation was performed on 10 beagles (age range: 20–24 months; weight range: 9–12 kg). Saline containing 1/80,000 epinephrine was injected intraorally in 1-mL increments into the nasal mucosa of the soft palate (n = 4), posterior pharyngeal wall (n = 3), or bilateral pharyngeal walls (n = 3) of each dog. Nasal air leakage was measured under rebreathing until velopharyngeal closure was achieved; the measurement was performed using flow meter sensors on both nasal apertures, and the oral cavity was filled with alginate impression material to prevent oral air leakage.

**Results:**

Pre-injection, the dogs exhibited an average of 0.455 L/s air leakage from the nasal cavity. The dogs with saline injected into the nasal mucosa of the soft palate achieved steady augmentation, and nasal air leakage disappeared under rebreathing following 6-mL saline injection. Conversely, nasal air leakage remained in the dogs with saline injected in the posterior pharyngeal wall or bilateral pharyngeal walls.

**Conclusions:**

During VPI treatment in dogs, augmentation was most effective at the nasal mucosa of the soft palate. Improvement in nasal air leakage was highly dependent on the saline injection volume. Although velopharyngeal structures vary between dogs and humans, velopharyngeal closure style is similar. Thus, our results may aid in the treatment of VPI patients.

## Introduction

Velopharyngeal closure between the oral and nasal cavities during speech is the most important function of the soft palate, to prevent the reflux of air and liquids into the nasopharynx during speech and swallowing, respectively. Velopharyngeal insufficiency (VPI), which is incomplete velopharyngeal closure, causes functional problems with speech including hypernasality, hyponasality, nasal turbulence, audible nasal emission, weak pressure consonants, and impaired speech intelligibility [1]. Some cleft palate patients exhibit VPI even after palatoplasty, thereby necessitating the use of several additional operation methods [2–3]. Traditionally, the velopharyngeal plasty using posterior pharyngeal wall flap is a standard operation method for VPI [4–6]; however, it is difficult to adapt this method for treatment in children, as it causes fundamental change to the velopharyngeal form, which may result in sleep apnea or an inability to perform nasal intubation during future orthodontic surgery [7–10].

Recently, several studies have reported that velopharyngeal structure augmentation methods, using implants or injectable materials, have served as alternatives to velopharyngeal plasty [11–23]. There are various artificial and biological materials that may serve in this capacity, including silicone, Teflon, porous polyethylene, Gore-Tex, calcium hydroxyapatite, auricular or costal cartilage, and autologous fat. The anatomic sites of implantation or injection also vary widely. Currently, surgeons perform augmentation based on their own empirically determined criteria as there are no standards for the use of a particular augmentation approach.

Among previous reports, only one study was an animal experimental trial; however, it merely served to investigate the histological fate of autogenous fat that was injected submucosally in the oropharyngeal region in rabbits [24]. Moreover, there have been no reports about optimal anatomic sites of implantation or injection.

In the present study, we experimentally investigated the optimal sites of velopharyngeal structure augmentation using saline injection in dogs. Dogs naturally exhibit VPI; thus, the rhinopharynx is not completely closed, even when the soft palate is lifted [25]. Previously, Kogo M et al. revealed that the levator veli palatini muscle (LVP), the principal muscle responsible for generating palatal movement, was active in expiration during hyperpnea occurring under rebreathing conditions [26]. Thus, we suspected that if velopharyngeal structure augmentation could lead to perfect velopharyngeal closure in dogs during rebreathing, this technique may be also useful for the treatment of patients with VPI.

## Materials and methods

Velopharyngeal structure augmentations using saline injection were performed at the Large Animal Laboratory of the Graduate School of Dentistry of Osaka University, using 10 beagles (TOYO beagle; Oriental Yeast Co., Tokyo, Japan) aged 20–24 months and weighed 9–12 kg. All dogs were housed in separate cages and provided solid food (Oriental Yeast Co., Tokyo, Japan) and water ad libitum. All experimental protocols were reviewed and approved by the Intramural Animal Care and Use Committee of Osaka University Graduate School of Dentistry (approval number is 28-011-0).

All procedures were performed under general anesthesia administered by an intramuscular injection of medetomidine (0.02 mg/kg) and midazolam (0.3 mg/kg) followed by an intraperitoneal injection of sodium pentobarbital (25 mg/kg) 15 minutes after the intramuscular injection of anesthesia. Animals were fixed in the supine position after the ventilation tube was passed through the mouth, and all efforts were made to minimize suffering.

**Fig 1:**
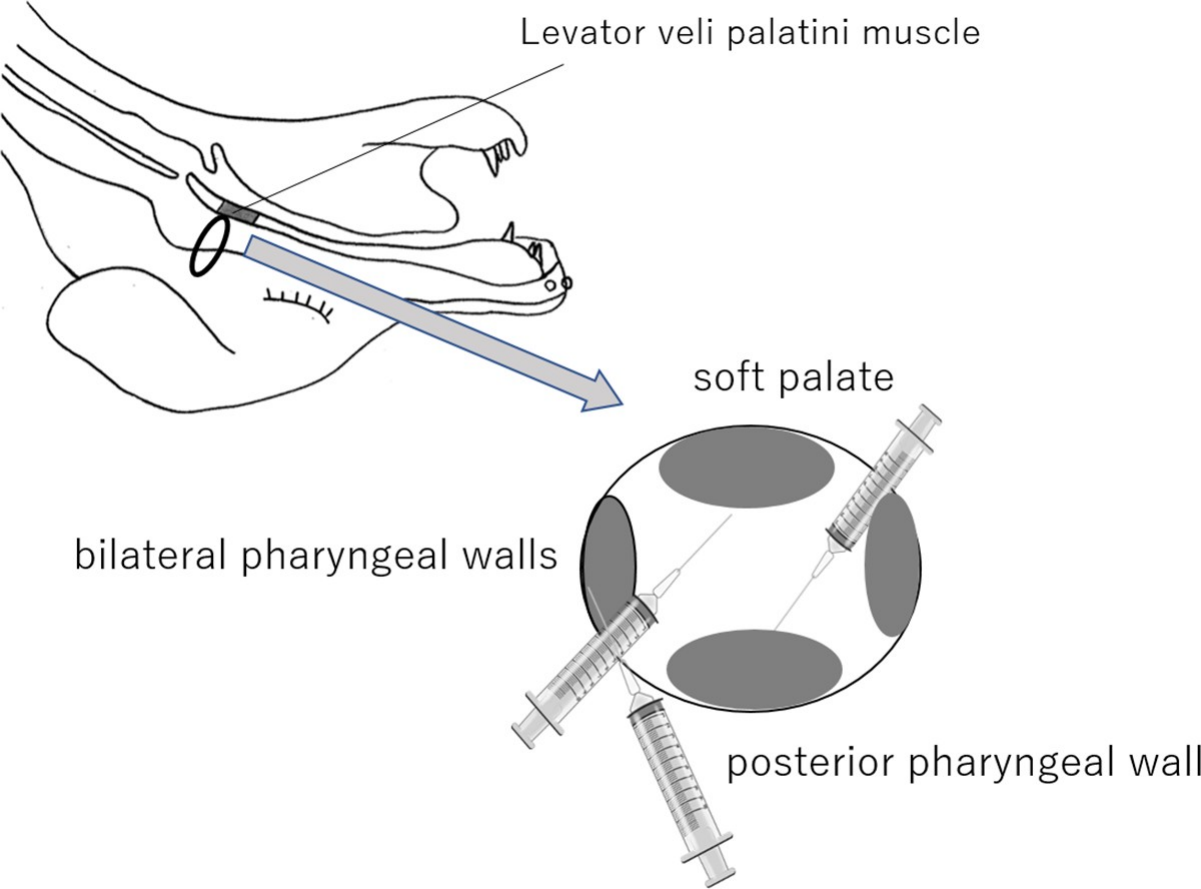
Injection sites around the velopharyngeal structure.

**Fig 2:**
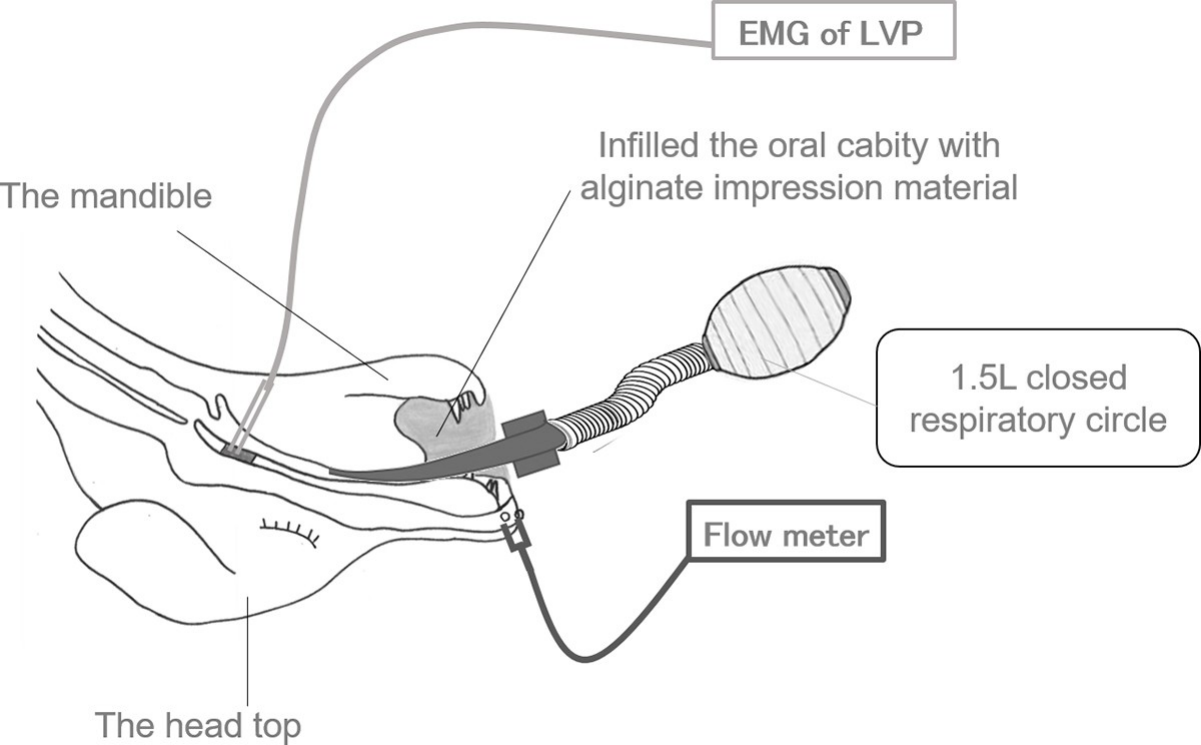
Schematic diagram of the experimental model.

We exposed the LVP using an intraoral approach without damaging the muscles; subsequently, two bipolar platinum-iridium wire-hook electrodes (polar distance: 2 mm, diameter: 50 μm) were implanted into the bilateral LVP in the visible field, and electromyographic (EMG) activities of the muscles were recorded with a bioelectric amplifier (DAM-50; Nihon Koden Kogyo Co., Japan). EMGs of the LVP were recorded to check LVP movement during rebreathing. Next, 1 mL of saline containing 1/80,000 epinephrine was injected intraorally into the nasal mucosal side of the anterior two-thirds of the soft palate (n = 4), the posterior pharyngeal wall (n = 3), or the bilateral pharyngeal walls (n = 3); each injection was checked using an endoscope (i-Vets 8.0; SCETI K., Tokyo, Japan) (Fig 1). The inside end of the ventilation tube was withdrawn from the trachea to the oral cavity, to allow expiration through the nasal cavity. Then, the oral cavity was infilled with alginate impression material except for the electrode and the ventilation tube, to prevent oral air leak. While the dogs were under the rebreathing system, the amount of air leakage from the nasal cavity was measured by a flow meter (TSD117; BIOPAC Systems Inc., Japan) using the rubber tubes connected to the flow meter’s sensor on the front of both nasal apertures (Fig 2). The outside of the rubber tubes was packed with quick self-curing acrylic resin (UNIFAST II; GC Co., Tokyo, Japan) to prevent air leakage. Data from the flow meter were recorded on a personal computer (U24a-px3210r Windows7; ASUSTek Computer Inc., Japan) using data acquisition and analysis software (Labchart7; AD Instruments, Japan) through a DC Amplifier (DA100C; BIOPAC Systems Inc., Japan), an Analog output module (HLT100-C; BIOPAC Systems Inc., Japan), and an AD converter (Power lab; AD Instruments Co., Tokyo, Japan).

The rebreathing system is one of the ways to get the load breathing, which leads to the movement of LVP. The rebreathing system is a 1.5-L closed airway circuit constructed by connecting the intraoral tube to the bag valve mask. The level of partial pressure of carbon dioxide was increased by complete rebreathing, and it leads the activity of the LVP, which is one of the accessory respiratory muscles [26]. In our study, respiration was spontaneous without artificial ventilation.

**Fig 3:**
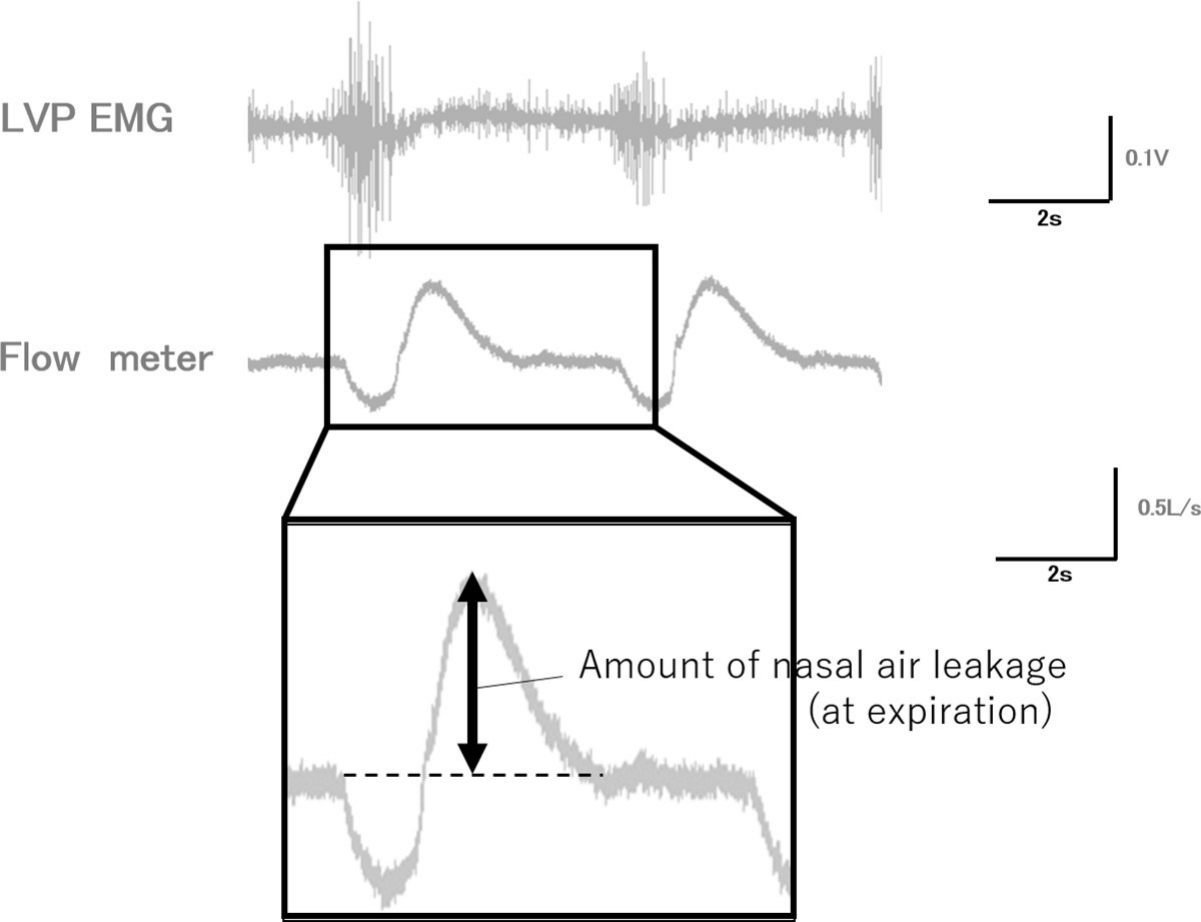
Measurement method of the amount of the nasal air leakage.

Saline was injected in 1-mL increments, and the augmentation was performed in a step-by-step manner. Nasal air leakage was measured for each size of augmentation during rebreathing until complete velopharyngeal closure was achieved. Nasal air leakage was measured using the EMG of the LVP, from the baseline to the peak of the waveform (Fig 3).

In addition, the increase rate of nasal resistance of expiration and inspiration was calculated when the saline was injected into the nasal mucosal site of the soft palate. The increase rate of nasal resistance was quantified with the following expression [27].

$$\begin{array}{l} Increase\;rate\;of\;nasal\;resistance=\frac{\left(R\left(x\right)-R\left(0\right)\right)}{R\left(0\right)}\\ \;=\frac{\left(\frac{V}{N\times F\left(x\right)}-\frac{V}{N\times F\left(0\right)}\right)}{\frac{V}{N\times F\left(0\right)}}\\ \;=\frac{F\left(0\right)}{F\left(x\right)}-1 \end{array}$$

(R(x): nasal resistance when x mL saline was injected, F(x): nasal air flow per 1 sec when x mL saline was injected, V: respiratory pressure, N: constant)

Normality of the data were evaluated and due to their nonparametric nature, data were analyzed using the Kolmogorov-Smirnov test and Kruskal-Wallis test.

## Results

The median amount of nasal air leakage in the all dogs before the saline injection was 0.82 L/sec. The dogs injected with saline on the nasal mucosa side of the soft palates achieved steady augmentation, and nasal air leakage disappeared under rebreathing with 6 mL saline injection (Table 1). Conversely, nasal air leakage remained in the dogs that underwent posterior pharyngeal wall injection even when saline was injected until the structural tensility reached its limit; this air leakage was nearly 35% of normal airflow (0.25 L/sec) after 6 mL saline injection. The dogs with saline injected in the bilateral pharyngeal walls did not show a steady decrease in air leakage. The injected saline leaked into the posterior pharyngeal cavity or the soft palate when the injection amount exceeded 4 mL and therefore, we did not inject a volume of more than 4 mL. In fact, the leakage from the bilateral pharyngeal walls increased by >1 mL, relative to the injection volume. It appeared that the flat pharyngeal cavity was transmuted into a square-like shape following saline injection into the bilateral pharyngeal walls.

**Table 1:** Relationship between median volume of nasal air leakage and volume of injected saline. The interquartile ranges are shown in parentheses.

		The amount of nasal air leakage (L/s)			P-value (Prob>ChiSq)
		soft palate	posterior pharyngeal wall	bilateral pharyngeal walls	
The amount of injected saline (ml)	**0**	0.84 (0.55–0.87)	0.75 (0.44–0.82)	0.64 (0.46–0.86)	0.399
	1	0.52 (0.37–0.88)	0.63 (0.35–0.68)	0.40 (0.31–0.50)	0.328
	2	0.46 (0.30–0.74)	0.63 (0.35–0.68)	0.52 (0.32–0.72)	0.904
	3	0.27 (0.24–0.65)	0.39 (0.35–0.54)	0.56 (0.38–0.88)	0.236
	4	0.23 (0.15–0.27)	0.35 (0.33–0.37)	0.56 (0.34–0.77)	0.030*
	5	0.15 (0.12–0.19)	0.30 (0.30–0.35)		0.034*
	6	0.00 (0.00–0.00)	0.25 (0.20–0.26)		0.019*

*p<0.05

The results show the means of experimental values in three or four dogs.

**Fig 4:**
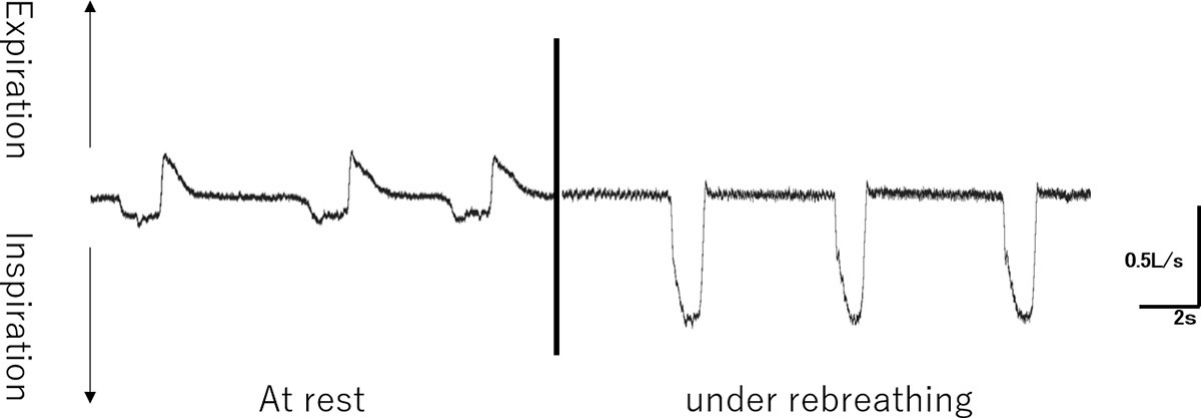
Nasal air flow with 6 mL saline injection to the soft palate.

Notably, the dogs with saline injected into the soft palate retained nasal air flow under normal breathing (at rest), even after undergoing maximum saline injection; moreover, inspiration under rebreathing was not prevented (Fig 4). The increase rate of nasal resistance was maximum at expiration depending on the volume of saline injection; however, the increase rate of nasal resistance at inspiration did not vary much with the volume of saline injection (Table 2).

**Table 2:** The median increase rate of nasal resistance by saline injection to the soft palate. The interquartile ranges are shown in parentheses.

		Increase rate of nasal resistance		P-value (Prob>ChiSq)
		Expiration	Inspiration	
The amount of injected saline (ml)	0	0.00 (0.00–0.00)	0.00 (0.00–0.00)	-
	1	0.50 (0.03–0.69)	0.33 (0.10–1.32)	0.734
	2	0.81 (0.19–0.96)	0.59 (0.49–1.52)	0.865
	3	1.43 (0.27–2.49)	0.37 (0.23–2.97)	0.807
	4	2.64 (2.06–2.85)	1.06 (0.83–1.45)	0.011*
	5	3.68 (3.23–5.01)	1.18 (1.05–1.63)	0.007*
	6		1.28 (1.24–1.98)	-

*p<0.05

## Discussion

In this study, we investigated velopharyngeal structure augmentation in dogs, as they demonstrate similar velopharyngeal physiological properties to humans, especially in terms of respiration patterns [24].

It is well known that the structure of the pharynx in humans is different from that in many animals. The pharynx of the humans is right-angled, and the epiglottis does not touch the velum whereas the pharynx of animals is straight, and the velum and the epiglottis can touch each other during breathing at rest. However, humans breathe exclusively through the nose at rest; during physical exercise, the breathing route is switched from nasal to oronasal in a manner that is also present in monkeys and dogs, and for this reason, we selected dogs as the experimental animals [28,29]. Many other animals breathe entirely through the nose. In humans, monkeys, and dogs, when hyperpnea is produced by the rebreathing technique, the LVP is active during the expiratory phase but inactive during the inspiratory phase, and this results in oronasal breathing [26,30–32]. In our study, this phenomenon was also observed; furthermore, the nasal air leakage increased during inspiration and decreased during expiration.

VPI occurs in 20–30% of patients after palatoplasty due to the shortfall of the original soft palate, scarring of the soft palate after palatoplasty, lack of movement of the LVP (important in lifting the soft palate), or paralysis of the LVP [1]. However, if VPI is successfully treated by speech therapy, a suitable bulb-attached palatal lift prosthesis (Bulb-PLP), and a velopharyngeal plasty, following correct diagnosis and linguistic evaluation, this condition could be substantially improved in many cases [1].

Velopharyngeal structure augmentation methods are used as a helpful alternative to velopharyngeal plasty, but these methods remain to be outside of the standard treatment approaches. Bishop A et al. reviewed reports of several past investigations on autologous fat grafting for the treatment of VPI; three of these reported grafting to the posterior pharyngeal wall, and eight of these reported simultaneous grafting to three regions: soft palate, posterior pharyngeal wall, and bilateral pharyngeal walls [21]. Importantly, questions remained regarding the optimal graft volume and injection sites. Recently, only one report discussed augmentation that was limited to the soft palate [23]. Furthermore, many reports on velopharyngeal augmentation have found that this treatment did not improve hypernasality in some VPI patients [13,15,18,19,21,23].

Our study evaluated the optimal injection sites for improvement of VPI, and we found that saline injection to the nasal mucosa of the soft palate is most effective in dogs. The advantage of our method is that it allows an adjustment of the injection volume corresponding to the level of nasal air leakage. To our knowledge, this study is the first short-term experiment to use saline and therefore, we cannot easily compare our results to those of previous studies. Further studies must include long-term evaluations of several materials such as autologous fat, costal cartilage, and silicone because saline is easy to absorb, and it is only a temporary filler.

During velopharyngeal plasty, the nasal resistance increased, and airway narrowing had occurred. However, the nasal resistance to the injection to the nasal mucosal side of the soft palate was not significantly increased during inspiration in contrast to that during expiration. It was considered that it was due to an increase in the tension of the mucous membrane by the injection, and it prevented the movement of the membrane during inspiration. Thus, injection to the soft palate is considered to have more advantages than velopharyngeal plasty.

Human and dog velopharyngeal structures are quite different whereas they may function similarly during respiration; we must re-evaluate the amount of injection when applying our results to human patients. Moreover, we cannot experimentally evaluate speech in dogs. Another limitation of our study is the small sample size. VPI does not vary widely across groups of normal dogs whereas the VPI in humans exhibits considerable individual variability resulting from various prior treatments and/or age-related changes. Finally, growth is an important factor. If the treatment for VPI is performed during childhood, VPI can reoccur after growth because velopharyngeal space will become wider. As a practical proposition, the ideal injection volume for a patient with VPI may be determined by lateral cephalic radiography, computed tomography, magnetic resonance imaging, or endoscopy. Lau D et al. assessed each patient individually via nasoendoscopy to determine the amount of fat to be grafted; in that study, approximately 1 mL of the fat graft was used for every 0.1 cm^2^ of VPI [20]. An alternate method to determine the amount of injection may include gradual injection while assessing speech, performed under local anesthesia.

Our study results clearly demonstrate that the augmentation of the nasal mucosa of the soft palate assists in the management of air leakage in dogs. Although it is unclear how extensively these findings will influence the treatment of patients with VPI, our present results certainly warrant additional examination.

## References

[1] Woo AS. Velopharyngeal dysfunction. Semin Plast Surg. 2012;26(4): 170–177. 10.1055/s-0033-1333882 24179450 PMC3706038

[2] Ruding R. Cleft palate: Anatomic and surgical considerations. Plast Reconstr Surg. 1964;33: 132–147. 14119663

[3] Brown AS, Cohen MA, Randall P. Levator muscle reconstruction: Does it make a difference? Plast Reconstr Surg. 1983;72(1): 1–8. 6867168 10.1097/00006534-198307000-00001

[4] Rogers C, Konofaos P, Wallace RD. Superiorly based pharyngeal flap for the surgical treatment of velopharyngeal insufficiency and speech outcomes. J Craniofac Surg. 2016;27(7): 1746–1749. 10.1097/SCS.0000000000003050 27763974

[5] Naran S, Ford M, Losee JE. What’s new in cleft palate and velopharyngeal dysfunction management? Plast Reconstr Surg. 2017;139(6): 1343e–1355e. 10.1097/PRS.0000000000003335 28538580

[6] Kogo M, Sakai T, Harada T, Nohara K, Isomura ET, Seikai T, et al Our unified pharyngeal flap operation. J Cleft Lip Palate Craniofac Anomal. 2017;4(3): 189–191.

[7] Orr WC, Levine NS, Buchanan RT. Effect of cleft palate repair and pharyngeal flap surgery on upper airway obstruction during sleep. Plast Reconstr Surg. 1987;80(2): 226–232. 3602172 10.1097/00006534-198708000-00010

[8] Valnicek SM, Zuker RM, Halpern LM, Roy WL. Perioperative complications of superior pharyngeal flap surgery in children. Plast Reconstr Surg. 1994;93(5): 954–958. 8134488 10.1097/00006534-199404001-00009

[9] Aboloyoun AI, Ghorab S, Farooq MU. Palatal lifting prosthesis and velopharyngeal insufficiency: preliminary report. Acta Med Acad. 2013;42(1): 55–60. 10.5644/ama2006-124.71 23735067

[10] Campos LD, Trindade-Suedam IK, Sampalo-Teixeira AC, Yamashita RP, Lauris JR, Lorenzi-Filho G, et al Obstructive sleep apnea following pharyngeal flap surgery for velopharyngeal flap surgery for velopharyngeal insufficiency: A prospective polysomnographic and aerodynamic study in middle-aged adults. Cleft Palate Craniofac J. 2016;53(3): e53–e59. 10.1597/14-152 25794015

[11] Brauer RO. Retropharyngeal implantation of silicone gel pillows for velopharyngeal incompetence. Plast Reconstr Surg. 1973;51(3): 254–262. 4266055 10.1097/00006534-197303000-00003

[12] Remacle M, Bertrand B, Eloy P, Marbaix E. The use of injectable collagen to correct velopharyngeal insufficiency. Laryngoscope. 1990;100(3): 269–274. 10.1288/00005537-199003000-00011 2308450

[13] Dejonckere PH, van Wijngaarden HA. Retropharyngeal autologous fat transplantation for congenital short palate: a nasometric assessment of functional results. Ann Otol Rhinol Laryngol. 2001;110(2): 168–172. 10.1177/000348940111000213 11219525

[14] Klotz DA, Howard J, Hengerer AS, Slupchynskj O. Lipoinjection augmentation of the soft palate for velopharyngeal stress incompetence. Laryngoscope. 2001;111(12): 2157–2161. 10.1097/00005537-200112000-00015 11802016

[15] Leuchter I, Schweizer V, Hohlfeld J, Pasche P. Treatment of velopharyngeal insufficiency by autologous fat injection. Eur Arch Otorhinolaryngol. 2010;267(6): 977–983. 10.1007/s00405-009-1157-7 20033195

[16] Leboulanger N, Blanchard M, Denoyelle F, Glynn F, Charrier JB, Roger G, et al Autologous fat transfer in velopharyngeal insufficiency indications and results of a 25 procedures series. Int J Pediatr Otorhinolaryngol. 2011;75(11): 1401–1407. 10.1016/j.ijporl.2011.08.001 21872348

[17] Cantarella G, Mazzola RF, Mantovani M, Baracca G, Pignataro L. Treatment of velopharyngeal insufficiency by pharyngeal and velar fat injections. Otolaryngol Head Neck Surg. 2011;145(3): 401–403. 10.1177/0194599811411655 21636838

[18] Filip C, Matzen M, Aagenæs I, Aukner R, Kjøll L, Høgevold HE, et al Speech and magnetic resonance imaging results following autologous fat transplantation to the velopharynx in patients with velopharyngeal insufficiency. Cleft Palate Craniofac J. 2011;48(6): 708–716. 10.1597/09-161 21463181

[19] Filip C, Matzen M, Aagenæs I, Aukner R, Kjøll L, Høgevold HE, et al Autologous fat transplantation to the velopharynx for treating persistent velopharyngeal insufficiency of mild degree secondary to overt or submucous cleft palate. J Plast Reconstr Aesthet Surg. 2013;66(3): 337–344. 10.1016/j.bjps.2012.11.006 23254179

[20] Lau D, Oppenheimer AJ, Buchman SR, Berger M, Kasten SJ. Posterior pharyngeal fat grafting for velopharyngeal insufficiency. Cleft Palate Craniofac J. 2013;50(1): 51–58. 10.1597/11-038 22329568

[21] Bishop A, Hong P, Bezuhly M. Autologous fat grafting for the treatment of velopharyngeal insufficiency: state of the art. J Plast Reconstr Aesthet Surg. 2014;67(1): 1–8. 10.1016/j.bjps.2013.09.021 24090720

[22] Filip C. Response re: Autologous fat grafting for the treatment of velopharyngeal insufficiency: state of the art. J Plast Reconstr Aesthet Surg. 2014;67(8): 1155–1156. 10.1016/j.bjps.2014.02.001 24581953

[23] Boneti C, Ray PD, Macklem EB, Kohanzadeh S, de la Torre J, Grant JH. Effectiveness and safety of autologous fat grafting to the soft palate alone. Ann Plast Surg. 2015;74: S190–S192. 10.1097/SAP.0000000000000442 25695441

[24] Canady JW, Thompson SA, Moon JB, Glowacki RL. Augmentation of oral tissues in rabbit using autogenous fat. Cleft Palate Craniofac J. 1995;32(1): 1–6. 10.1597/1545-1569_1995_032_0001_aootir_2.3.co_2 7727481

[25] Adachi T, Kogo M, Iida S, Hamaguchi M, Matsuya T. Measurement of velopharyngeal movement induced by isolated stimulation of levator veli palatini and pharyngeal constrictor muscles. J Dent Res. 1997;76(11): 1745–1750. 10.1177/00220345970760110501 9372791

[26] Kogo M, Kurimoto T, Koizumi H, Nishio J, Matsuya T. Respiratory activities in relation to palatal muscle contraction. Cleft Palate Craniofac J. 1992;29(2): 174–178. 10.1597/1545-1569_1992_029_0174_rairtp_2.3.co_2 1571352

[27] Kodama A. The sensation of nasal obstruction and nasal airway patency. Practica Oto-Rhino-Laryngologica. 1983;76(10): 2671–2676. (In Japanese)

[28] Rodenstein DO, Stănescu DC. Soft palate and oronasal breathing in humans. J Appl Physiol Respir Environ Exerc Physiol. 1984;57(3): 651–657. 10.1152/jappl.1984.57.3.651 6490454

[29] Rodenstein DO, Stănescu DC. The soft palate and breathing. Am Rev Respir Dis. 1986;134(2): 311–325. 10.1164/arrd.1986.134.2.311 3527007

[30] Koizumi H, Kogo M, Matsuya T. Effect of lung inflation on levator veli palatini muscle activity. J Dent Res. 1995;74(5): 1235–1239. 10.1177/00220345950740051401 7790602

[31] Koizumi H, Kogo M, Matsuya T. Coordination between palatal and laryngeal muscle activities in response to rebreathing and lung inflation. Cleft Palate Craniofac J. 1996;33(6): 459–462. 10.1597/1545-1569_1996_033_0459_cbpalm_2.3.co_2 8939368

[32] Kogo M, Tanaka S, Ishii S, Hamaguchi M, Iida S, Matsuya T. Activities of superior pharyngeal constrictor and levator veli palatini muscles related to respiration in dogs. Cleft Palate Craniofac J. 1997;34(4): 338–341. 10.1597/1545-1569_1997_034_0337_aospca_2.3.co_2 9257025

